# Genetic dissection of the impact of lncRNA AI662270 during the development of atherosclerosis

**DOI:** 10.1186/s12967-023-03962-6

**Published:** 2023-02-08

**Authors:** Yang Hong, Yue Zhang, Hui Chen, Xueqing Tang, Hongrui Zhao, Ziyu Meng, Xueling Jia, Wenfeng Liu, Xiaohan Li, Lin Wang, Xinrui Zhong, Xuefeng Bai, Heyang Sun, Philipp Kopylov, Bestavashvili Afina, Dmitry Shchekochikhin, Yong Zhang, Xin Liu, Yuhua Fan

**Affiliations:** 1 Department of Pathology and Pathophysiology, College of Basic Medical Sciences, Harbin Medical University-Daqing, Daqing, 163319 Heilongjiang People’s Republic of China; 2 Department of Pharmacology, State-Province Key Laboratories of Biomedicine-Pharmaceutics of China, Key Laboratory of Cardiovascular Medicine Research, Ministry of Education, College of Pharmacy, Harbin Medical University, Harbin, 150081 Heilongjiang People’s Republic of China; 3 School of Medical Informatics, Harbin Medical University, Daqing Campus, Daqing, 163319 People’s Republic of China; 4 Department of Preventive and Emergency Cardiology, Sechenov First Moscow State Medical University, Moscow, Russian Federation; 5 Department of Cardiology, Functional and Ultrasound Diagnostics, N.V. Sklifosofsky, I. M. Sechenov First Moscow State Medical University, Moscow, Russian Federation

**Keywords:** AI662270, Abca1, Cholesterol efflux, Foam cell formation, Atherosclerosis

## Abstract

**Background:**

Atherosclerosis is driven by synergistic interactions between pathological biomechanical and lipid metabolic factors. Long noncoding RNAs (LncRNAs) have been implicated in atherogenesis. The purpose of this study was to investigate the potential mechanism of lncRNA AI662270 on macrophage cholesterol transport in atherosclerosis.

**Methods:**

*Apolipoprotein E* deficiency (*ApoE*^−/−^) mice were fed a high fat diet for 16 weeks to construct atherosclerotic model, and the mice were injected with recombinant lentivirus carrying AI662270 gene to overexpress AI662270. Macrophages were cleared by liposomal clondronate in vivo. Fundamental experiments and functional assays, hematoxylin and eosin staining, oil red O staining and others, were performed to evaluate the function of AI662270 on atherogenesis. Peritoneal macrophages were treated with oxidized low density lipoprotein (ox-LDL) to simulate in vitro model. Mechanism assays, RNA-interacting protein immunoprecipitation, RNA–protein pulldown and others, were performed to study the regulatory mechanism of AI662270 in macrophages.

**Results:**

The novel AI662270 was mainly enriched in macrophages, but not in endothelial cells, smooth muscle cells and fibroblasts of mouse atherosclerotic lesions and was upregulated by ox-LDL. Overexpression of AI662270 resulted in lipid accumulation, larger atherosclerotic plaques and cardiac dysfunction in vivo. After macrophages were removed, the pro-atherogenic effect of AI662270 disappeared. Downregulation of AI662270 in macrophages protected against foam cell formation by potentiating cholesterol efflux and reducing intracellular total cholesterol. The opposite effect was observed in macrophage-specific AI662270-overexpressed cells in vitro. AI662270 bound to adenosine triphosphate-binding cassette transporter A1 (Abca1) responsible for regulating cholesterol efflux in macrophages. Forced expression of AI662270 in macrophages decreased Abca1 expression. The reverse occurred when expression of AI662270 was repressed.

**Conclusion:**

These findings reveal an essential role for AI662270 in atherosclerosis progression by regulating cholesterol efflux from macrophages.

**Supplementary Information:**

The online version contains supplementary material available at 10.1186/s12967-023-03962-6.

## Background

Atherosclerosis is a chronic inflammatory process characterized by complex interactions among modified lipoproteins, foam cells, or monocyte-derived macrophages, and dysfunction of endothelial cells and smooth muscle cells [1–3]. Macrophages are crucial in plaque occurrence, growth, and ultimate rupture [4]. The underlying pathology is partially ascribed to the engulfment of oxidized low density lipoprotein (ox-LDL) by macrophages to produce cholesterol-laden foam cells, a hallmark of atherosclerosis. Subsequent production of proinflammatory cytokines protects against matrix metalloproteinase degradation, leading to lesion formation and arterial plaque rupture [5, 6]. Adenosine triphosphate-binding cassette transporter A1 (Abca1) is responsible for cholesterol efflux in macrophages [7]. Either deficiency or downregulation of Abca1 can increase the number of foam cells to aggravate atherogenesis [8, 9]. Thus, Abca1 established pivotal role in the regulation of atherogenesis (Additional file 1).

Long noncoding RNAs (LncRNAs) are RNA transcripts longer than 200 nt with no protein-coding capacity [10]. Increasing evidences have documented that lncRNAs are involved in regulating multiple cellular/molecular processes including chromatin modification, RNA stability, alternative splicing, cell metabolism, which are closely related to the pathophysiological processes of atherosclerosis [11, 12]. Hematopoietic deficiency of lncRNA MALAT1 enhances monocytes adhesion to endothelial cells and increases the content of proinflammatory mediators, thereby promoting atherosclerotic lesion formation [13]. Deletion of lncRNA MeXis can alter chromosome architecture at the *Abca1* locus and impede cholesterol efflux to accelerate atherogenesis in mice [14]. Increased expression of lncRNA MIAT is linked to conditions involved in attenuation of efferocytosis in advanced atherosclerosis [15]. Dysregulated expression of many lncRNAs has been described with potential connection to the development of atherosclerosis, such as lncRNA GAS5 [16]. However, their characterization remains unclear, prompting the present exploration of the potential roles of novel lncRNAs in atherogenesis.

LncRNA AI662270 was originally identified by Shibata et al*.* [17], who demonstrated its upregulation upon exposure to ischemia reperfusion. Mikiko et al*.* recently elucidated that AI662270 could be controlled by the lysine methyltransferase G9a, thereby influencing the G1 phase of the cell cycle [18]. Here, we report that lncRNA AI662270, which is enriched in the plaque, might be implicated in promoting atherogenesis.

## Methods

### Mice

Male *apolipoprotein E* deficiency (*ApoE*^*−/−*^) mice and male C57BL/6 mice (Wild-Type, WT) were purchased from Nanjing Junke Biological Engineering Co., Ltd. and housed under standard housing conditions (temperature 23 ± 1 °C, humidity 55–60%). To explore the effect of AI662270 on atherosclerosis progression, 8-week-old male mice (22–25 g) were randomly divided into 4 groups: chow diet (CD) group, high fat diet (HFD) group, HFD + lentiviral vectors (Lv)-empty vector (null), and HFD + Lv-AI662270. The mice were fed with HFD containing 0.3% cholesterol and 21% (wt/wt) fat for 16 weeks. Simultaneously, the mice were also injected with 100 μL recombinant lentivirus carrying AI662270 gene (1 × 10^8^ UT/mL) to overexpress AI662270 (Lv-AI662270) or an equal volume of negative control (1 × 10^8^ UT/mL) (Lv-null) through the tail vein. After 16 weeks of management, mice (24 weeks old) were anesthetized and euthanized for assessing aortic lesion size and biochemical analysis. In order to verify that AI662270 affects atherosclerosis process by regulating macrophage function, liposomal clondronate (LC) was used to clear macrophage in vivo [19]. Mice were randomly divided into 4 groups: Lv-null + HFD, Lv-AI662270 + HFD, Lv-null + CL + HFD, Lv-AI662270 + CL + HFD. The mice were fed with HFD for 16 weeks. And mice were injected with CL intravenously to clear macrophages, 250 μL of LC (4 mg/mL) first and 125 μL every 4 days thereafter. Recombinant lentivirus Lv-AI662270 or Lv-null injection was given 2 days after the first LC injection. After 16 weeks, the mice were euthanized for follow-up experiments. All procedures were approved by the Institutional Animal Care and Use Committee of Harbin Medical University (Protocol (2009) -11). The use of animals complied with the Guide for the Care and Use of Laboratory Animals published by the United States National Institutes of Health (NIH Publication No. 85–23, revised 1996).

### Echocardiographic examination of heart

Anesthetized mice were positioned supine on the operating plate of the ultrasound instrument Vevo® 1100 (FUJIFILM VisualSonics, Tokyo, Japan). Cardiac function indicators including heart rate (HR), left ventricular internal dimension (LVID) and left ventricular posterior wall (LVPW) thickness were collected in systole and diastole, and ejection fraction (EF) and fractional shortening (FS) were calculated.

### Histology and morphometric analyses

Isolated hearts were perfused with 20 mL phosphate buffered saline (PBS; Solarbio) and then placed to 4% paraformaldehyde (PFA) for 24 h. Then, the hearts were immersed in 30% sucrose overnight. Finally, the hearts were coated in optimal cutting temperature compound (OCT) and kept at − 80 °C for future experiments. Serial sections of 6-μm thickness were cut with a cryostat. Histology, immunohistochemistry staining and morphometric measurements were performed as previously described [20]. Every fifth slide from the serial sections was stained with hematoxylin and eosin (H&E). Each consecutive slide was confronted with oil red O for detection of atherosclerotic lesion area, and stained by MASSON to assess collagen content. Transmission electron microscopy was employed to evaluate the foam cells formation in the lesions.

### *En face* oil red O staining

Thirty-five milliliters of oil red O stock solution (0.2% weight/volume in methanol) were mixed with 10 mL NaOH (1 M). Aorta was removed and cleaned with 78% methanol, followed by staining with 0.16% oil red O working solution for 50 min and rinse in 78% methanol for 3 min. Finally, the lesion area was analyzed as a percentage of the oil red O-stained area in the total aorta area.

### Fluorescence in situ hybridization (FISH)

To visualize the cellular distribution and expression alteration of AI662270, FISH was performed using a commercial kit (RiboBio, Guangzhou, China) on frozen sections of aortic root and cultured peritoneal macrophages. Briefly, samples were fixed and permeabilized in PBS containing 0.5% Triton X-100. Hybridization was performed overnight in a humidified chamber at 37 °C in the dark. All images were observed using a model FV1000 confocal microscope (Olympus, Tokyo, Japan). Cy3 (hybridization probes) and 4’,6-diamidino-2-phenylindole (DAPI) channels were used to measure the signals. Immunofluorescence staining was performed according to the manufacturer’s instructions. Antibody to Abca1 (ab18180, 1:150; Abcam, Cambridge, MA, USA) was used.

### Cell culture

Peritoneal macrophages from adult male WT mice were collected by peritoneal lavage 4 days after intraperitoneal (i.p.) injection of thioglycollate (3% w/v). Cells were cultured in DMEM supplemented with 10% fetal bovine serum (FBS). Non-adherent cells were removed, and macrophages were kept in fresh DMEM with 20% FBS and 20% L929-cell conditioned medium for 4 days [21]. Peritoneal macrophages were loaded with or without ox-LDL (100 µg/mL), followed by oil red O staining according to the manufacturer’s instructions (Nanjing Jiancheng Bioengineering Institute, China). The other cells, including mouse aortic endothelial cells (MAECs), mouse aortic vascular smooth muscle cells (MOVAS), and mouse fibroblasts L929, were cultured in a manner consistent with this.

### Cell counting kit-8 (CCK-8) assay

CCK-8 (Dojindo, Kumamoto, Japan) was used for the measurement of cell proliferation. Peritoneal macrophages were seeded in a 96-well plate and incubated with or without ox-LDL (100 µg/mL). After that, 10 µL CCK-8 solution were added to 100 µL cell culture medium and incubated for 1 h. Absorbance at 450 nm was detected using a plate reader.

### Synthesis and transfection of AI662270 constructs for knockdown and overexpression

AI662270 specific small interfering RNA (siRNA; siAI662270) and a negative control siRNA (siNC) were commercially synthesized by RiboBio. The constructs were transfected into cells for AI662270 knockdown at a final concentration of 150 nM. AI662270 cDNA was inserted into the pCDNA3.1 (Invitrogen, Carlsbad, CA, USA; AI662270-P with “P” indicating plasmid). Plasmid vectors (AI662270-P and empty vectors as a negative control construct NC-P) were transfected into cells for AI662270 overexpression at a final concentration of 2.5 μg/mL. Transfection was carried out using X-tremeGENE Transfection Reagent (#38906100; Roche, Basel, Switzerland) according to the manufacturer’s instructions. Macrophages were cultured in 6-well plates to 60% confluence and washed with serum-free medium before transfection. The macrophages were collected 48 h after transfection for subsequent experiments. The sequences of siAI662270-1 were: forward 5’-GGCAAUAUCACAAGUUGGUdTdT-3’ and reverse 5’-ACCAACUUGUGAUAUUGCCdTdT-3’; siAI662270-2: forward 5’-CGGCAUGGAAGUUUCUUCUdTdT-3’ and reverse 5’-AGAAGAAACUUCCA UGCCGdTdT-3’; siAI662270-3: forward 5’-GCUGUAGAUUGCUGGUAAUdTdT-3’ and reverse 5’-AUUACCAGCAAUCUACAGCdTdT-3’.

### RNA isolation and quantitative real-time reverse transcription polymerase chain reaction (qRT-PCR)

Total RNA from tissue or peritoneal macrophages was isolated using TRIzol reagent (Invitrogen) according to the manufacturer’s protocol. Total RNA (0.5 μg) was reverse transcribed to cDNA using the High-Capacity cDNA Reverse Transcription Kit (#FSQ-101; Toyobo, Osaka, Japan). SYBR Green PCR Master Mix Kit (#FSQ-301; Toyobo) was used for qRT-PCR to quantify the RNA levels of lncRNAs and mRNAs. qRT-PCR was performed on a 7500 FAST Real-Time PCR System (Applied Biosystems, Waltham, MA, USA) for 40 cycles. Glyceraldehyde 3-phosphate dehydrogenase (*Gapdh*) was used as housekeeping gene in qRT-PCR for relative level calculation of target RNA. The primer pair used for amplification of lncRNAs and mRNAs by qRT-PCR was showed in Table 1.

**Table 1:** Sequences of primers showed as follows

Primer name	Sequences (5’-3’)
AI662270	Forward: 5’-TTGAGCTAGACCCTTTTGATGTCA-3’ Reverse: 5’-TGTCAGGAATCTGAGGGTAAGATG-3’
Abca1	Forward: 5’-AAAACCGCAGACATCCTTCAG-3’ Reverse: 5’-CATACCGAAACTCGTTCACCC-3’
Abcg1	Forward: 5’-CTTTCCTACTCTGTACCCGAGG-3’ Reverse: 5’-CGGGGCATTCCATTGATAAGG-3’
CD36	Forward: 5’-TTGTACCTATACTGTGGCTAAATGAGA-3’ Reverse: 5’-CTTGTGTTTTGAACATTTCTGCTT-3’
SR-BI	Forward: 5’-GCCCATCATCTGCCAACT-3’ Reverse: 5’-TCCTGGGAGCCCTTTTTACT-3’
TNF-α	Forward: 5’-ACTCCAGGCGGTGCCTATGT-3’ Reverse: 5’-GTGAGGGTCTGGGCCATAGAA-3’
ARG-1	Forward: 5’-CTCCAAGCCAAAGTCCTTAGAG-3’ Reverse: 5’-AGGAGCTGTCATTAGGGACATC-3’
Retnla	Forward: 5’-CTGGGTTCTCCACCTCTTCA-3’ Reverse: 5’-TGCTGGGATGACTGCTACTG-3’
IL-1β	Forward: 5’-GCAACTGTTCCTGAACTCAACT-3’ Reverse: 5’-ATCTTTTGGGGTCCGTCAACT-3’
GAPDH	Forward: 5’-TGTGAGGGAGATGCTCAGTG-3’ Reverse: 5’-TGTTCCTACCCCCAATGTGT-3’

### Western blot analysis

Total protein was isolated from peritoneal macrophages using the same procedure described previously [22]. Protein concentration was measured using a BCA Protein Assay kit (Beyotime Biotechnology, Jiangsu, China). Equal amounts of protein lysates were separated by SDS-PAGE and transferred to nitrocellulose membranes (Millipore, Billerica, MA, USA), followed by blocking with 5% skimmed milk at room temperature for 1 h. Subsequently, the membrane was incubated with the primary antibodies against Abca1 (ab18180, 1:1000; Abcam, Cambridge, MA, USA) and GAPDH (1:2000; Proteintech, Rosemont, IL, USA) at 4 °C overnight. After rinsing with PBS containing Tween for three times, the membrane was incubated with fluorescence-conjugated anti-rabbit IgG secondary antibody (1:10,000; Jackson Immuno Research, West Grove, PA, USA). Western blot bands were obtained by using the Odyssey Infrared Imaging System (LI-COR Biosciences, Lincoln, NE, USA) and quantified with Odyssey v1.2 software (LI-COR Biosciences, Lincoln, NE, USA) by measuring the band intensity (area × OD) in each group and normalizing to GAPDH as an internal control.

### Immunofluorescence

Immunofluorescent staining was performed as previously described [23].Briefly, aorta roots were fixed with 4% PFA followed by permeabilization with 0.4% Triton X-100. Then, the tissue samples were blocked with 5% goat serum. Macrophages in plaques were probed by incubation with macrophage marker CD68 (1:200; MCA1957, AbD Serotec BioRad), Abca1 (ab18180, 1:200; Abcam, Cambridge, MA, USA). The vascular endothelium was labeled with the endothelial cell marker CD31 and co-stained with endothelial NOS (eNOS) (32027S, 1:200, Cell Signaling Technology, Boston, MA, USA) or inducible nitric oxide synthase (iNOS) (ab178945, 1:250; Abcam, Cambridge, MA, USA), respectively. Then, the samples were probed with florescence-labeled secondary antibody (1:500) and examined under a confocal laser scanning microscope (FV300, Olympus, Japan).

### RNA-interacting protein immunoprecipitation (RIP) and RNA–protein pulldown

RIP was performed using the Magna RIP™ RNA-Binding Protein Immunoprecipitation Kit (Cat#17–701; Millipore, Darmstadt, Germany) as described previously and according to the manufacturer’s instructions. RNA was reverse transcribed to cDNA using the ReverTra Ace qPCR RT Kit (#FSQ-101; Toyobo). The SYBR Green PCR Master Mix Kit (#FSQ-301; Toyobo) was used for qRT-PCR of the target genes using the 7500 Fast Real Time PCR system (Applied Biosystems). The cDNA of AI662270, which was cloned into pCDNA3.1 vector (Invitrogen). RNA for in vitro experiments was transcribed using the T7 RNA Polymerase Kit (Cat#D7069; Beyotime, Shanghai, China) according to the manufacturer’s instructions. The transcripts were labeled by RNA 3’ End Desthiobiotinylation Kit (Cat#20163; Oshkosh, WI, USA) according to the manufacturer’s instructions. The Pierce™ Magnetic RNA–Protein Pull-Down Kit (Cat#20164; Pierce) was used in RNA–protein pulldown experiments according to the manufacturer’s instructions. Biotin-labeled AI662270 (AI662270-1: full length; AI662270-2; AI662270-3; GenePharma) was used to transfect peritoneal macrophages using Lipofectamine 3000. AI662270-2: 5’-AGUUUGUGGAUUGAAAGGGAGGCAGAGCC-3’; AI662270-3: 5’-GGGCAGAAUCUUCUUGGAGCGUCCAGAAU-3’. The above two probe sequences were designed according to the database of catRAPID (Additional file 4: Fig. S3). M-280 streptavidin magnetic beads (Invitrogen) was used to incubate cell lysates. The protein pulled down by AI662270 was measured by Western blot with an anti-Abca1 antibody (ab18180, 1:1000; Abcam, Cambridge, MA, USA).

### Cholesterol efflux assay

Cholesterol efflux was measured using a cholesterol efflux assay kit (ab196985; Abcam). Peritoneal macrophages were incubated with or without the plasmid vector AI662270-P) and AI662270 specific siRNA (siAI662270) as required. The cells were treated with either HDL (50 µg/mL) or ApoA-I (10 µg/mL). The percentage of cholesterol efflux was that the value assessed with fluorescence intensity of media was divided by the value calculated with fluorescence intensity of cell lysate plus fluorescence intensity of media × 100%.

### Statistical analyses

Statistical comparisons among multiple groups were performed using analysis of variance (ANOVA) followed by Tukey test. Student’s *t*-test was performed for comparisons between the two groups. Two-tailed *p* < 0.05 indicates a statistically significant difference. All quantitative data are expressed as the mean ± SEM.

## Results

### AI662270 is highly expressed during the progression of atherosclerosis and enhances the lipid accumulation

To investigate the role of lncRNA in atherosclerosis, the mouse aortic gene expression profile (GSE2372: 3 *ApoE*^−/−^ mice samples, 3 normal samples) was downloaded from the Gene Expression Omnibus (GEO) database [24]. The differential expression genes were obtained by *p* value restriction (*p* < 0.05). The lncRNAs annotated here refer to Guo Xingli et al*.* [25] (Table 2). The results were consisted of 28 lncRNAs and 1196 genes. For further analysis, we constructed the co-expression networks identification by SubpathwayMiner and random walk [26]. Finally, we determine AI662270 through NONCODE database filtering [27] (Table 3).

**Table 2:** The ranks of lncRNAs in co-expression networks

Symbol	Entrzy ID	Weight	Score	Locations
4833427F10Rik	74601	0	0.00277876957377901	181
AI662270	100043636	0	0.00251577106035904	184
C130080G10Rikc	100303644	0	0.00225736637295161	187
5830444B04Rik	641454	0	0.00188583487717181	190
4933413J09Rik	71106	0	0.00124483172568078	205
E230016K23Rik	100504464	0	0.00122724566348287	207
9630028H03Rik	320684	0	0.00116053468059911	209
Gm11651	100379610	0	0.000887336768668902218	218
Med9os	100504121	0	0.000651206061114021258	258
Alkbh3os1	70852	0	0.0005347692944609	290
AI847159	100048701	0	0.000489479181151995293	293

**Table 3:** The lncRNAs expression in mouse tissues of NONCODE

Symbol	Entrzy ID	Heart	Hippocampus	Liver	Lung	Spleen	Thymus
4833427F10Rik	74601	×	×	×	0.0239257	0.03509	0.00895
AI662270	100043636	5.7258e−07	×	×	8.4978e−07	7.0881e−07	4.2673e−07
C130080G10Rikc	100303644	×	×	×	0.0137385	0.0426536	0.0101758

**Fig. 1:**
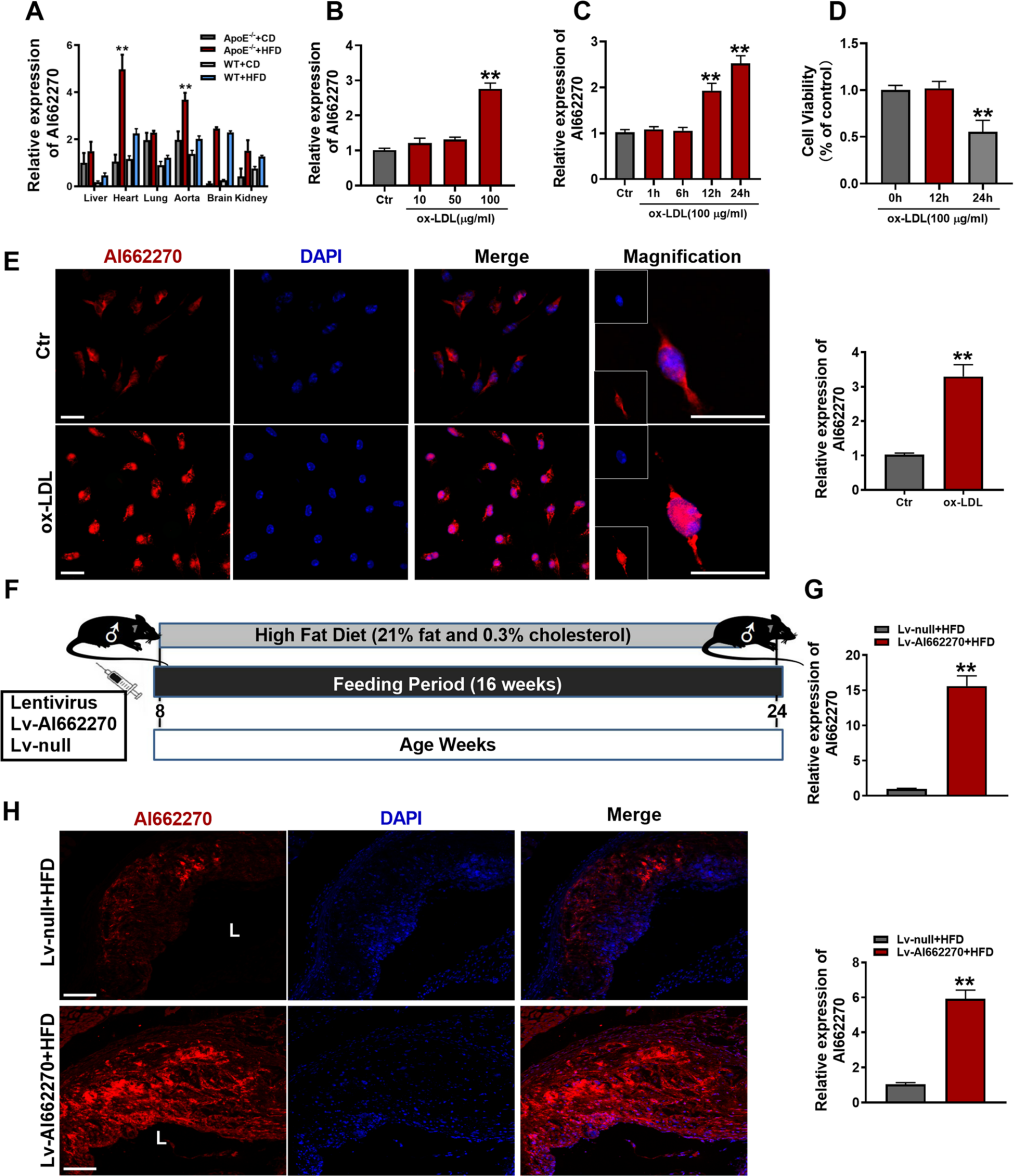
AI662270 is enriched in macrophages during atherogenesis. **A** Relative expression of AI662270 in various tissues of *ApoE*^−/−^ or WT mice with high fat diet (HFD) or chow diet (CD) (n = 6). **B, C** The effect of ox-LDL on AI662270 was confirmed by qRT-PCR (n = 6). **D** Cell counting kit-8 (CCK-8) was used to detect the effect of ox-LDL (100 µg/mL) on peritoneal macrophages (n = 6). **E, H** FISH was performed to monitor the expression and location of AI662270 in peritoneal macrophages (**E**) and aortic sinus (**H**), respectively (Scale bar = 20 μm, n = 6 for peritoneal macrophages; Scale bar = 200 μm, n = 3 for aortic sinus), “L” represents lumen. **F** Schematic representation of experimental mouse design. Briefly, 8-week-old male *ApoE*^−/−^ mice fed with HFD were intravenously injected with 100 μL recombinant lentivirus AI662270 (Lv-AI662270, 1 × 10^8^ UT/mL) or empty vector (Lv-null, 1 × 10^8^ UT/mL) for 16 weeks. **G** Transfection efficacy of Lv-AI662270 in aortas was measured by qRT-PCR (n = 6). **p < 0.01 *vs.* mice in CD or Lv-null treated mice in HFD in vivo, and Control (Ctr) in vitro. Data are expressed as mean ± SEM

To determine the potential mechanisms of AI662270 in atherosclerosis, *ApoE*^*−/−*^ and WT mice were fed with HFD for 16 weeks as atherosclerosis model in vivo, *ApoE*^*−/−*^ and WT mice were fed with CD as control group, respectively. We analyzed the expression levels of AI662270 in various organs of mice, and found that compared with CD-fed *ApoE*^*−/−*^ mice, AI662270 was highly expressed in aortas and hearts of *ApoE*^*−/−*^ mice fed with HFD for 16 weeks (Fig. 1A). In addition, incubation with ox-LDL (100 μg/mL) for 24 h increased the expression of AI662270 in peritoneal macrophages isolated from WT mice (Fig. 1B). Further examination, after incubation with ox-LDL (100 μg/mL) for 1, 6, 12, and 24 h, AI662270 increased in a time-dependent manner in peritoneal macrophages (Fig. 1C). However, CCK-8 assay showed that ox-LDL (100 μg/mL) incubation for 24 h dominantly reduced the viability of isolated peritoneal macrophages, while incubation with ox-LDL (100 μg/mL) for 12 h had little effect on its viability (Fig. 1D). Thus, the peritoneal macrophages were treated with ox-LDL (100 μg/mL) for 12 h in the following in vitro experiments. In addition, we found that AI662270 expression was not significantly changed in other cell types, including MAECs, MOVAS, and mouse fibroblasts L929, except in macrophages after treatment with ox-LDL. At the same time, compared with macrophages, the expression level of AI662270 in other cell types was quite low (Additional file 2: Fig. S1A). To further verify the effect of AI662270 on endothelial cell function, the protein expression levels of eNOS and iNOS were detected in vivo and in vitro, and it was found that their expression levels were not affected by AI662270 in vascular endothelium of mice fed with HFD (Additional file 2: Fig. S1B, C) and in MAEC treated with ox-LDL (Additional file 2: Fig. S1D, E), indicating that AI662270 had no significant effect on the function of vascular endothelial cells in atherosclerotic mice. In Fig. 1E, FISH examination showed that ox-LDL increased AI662270 expression level in peritoneal macrophages from WT mice, and AI662270 was evenly distributed in both the cytosol and nuclei of cells, which laid a foundation for us to explore the function and mechanism of AI662270.

In order to further explore the effect of AI662270 on atherosclerosis, 8-week-old athero-prone *ApoE*^*−/−*^ mice were randomly divided into 2 groups: *ApoE*^*−/−*^ mice intravenously injected with recombinant lentivirus vector expressing AI662270 (Lv-AI662270) and empty vector (Lv-null), and fed with HFD for 16 weeks (Fig. 1F). Meanwhile, *ApoE*^*−/−*^ mice were fed with CD as a control group. The high infection efficiency of Lv-AI662270 was confirmed by the remarkable increase in AI662270 levels in aortas (Fig. 1G). FISH analysis also demonstrated the significant increase of AI662270 in atherosclerotic plaques in HFD-fed mice infected with Lv-AI662270, compared to HFD-fed mice infected with Lv-null (Fig. 1H). Further experiment revealed that Lv-AI662270 infection significantly increased the plasma levels of total cholesterol (TC) and low density lipoprotein (LDL), and reduced high density lipoprotein (HDL), with no significant effect on triglyceride (TG) in *ApoE*^*−/−*^ mice fed with HFD. However, Lv-AI662270 infection had no statistically significant influence on lipid profiles and TG in *ApoE*^*−/−*^ mice fed with CD (Additional file 3: Fig. S2A–D). Moreover, although the results indicated that AI662270 overexpression slightly increased body weight, there was no obvious difference between Lv-AI662270 and Lv-null treated mice throughout the study (Additional file 3: Fig. S2E). On the other hand, as revealed by insulin tolerance test (ITT), *ApoE*^*−/−*^ mice infected with Lv-AI662270 and fed with HFD for 16 weeks had elevated glucose concentration and blunted responsiveness to insulin (Additional file 3: Fig. S2F), suggesting impaired insulin sensitivity. However, overexpression of AI662270 did not significantly affect glucose homeostasis in glucose tolerance test (GTT) (Additional file 3: Fig. S2G, H).

### AI662270 enlarges atherosclerotic lesion in *ApoE*^*−/−*^ mice

**Fig. 2:**
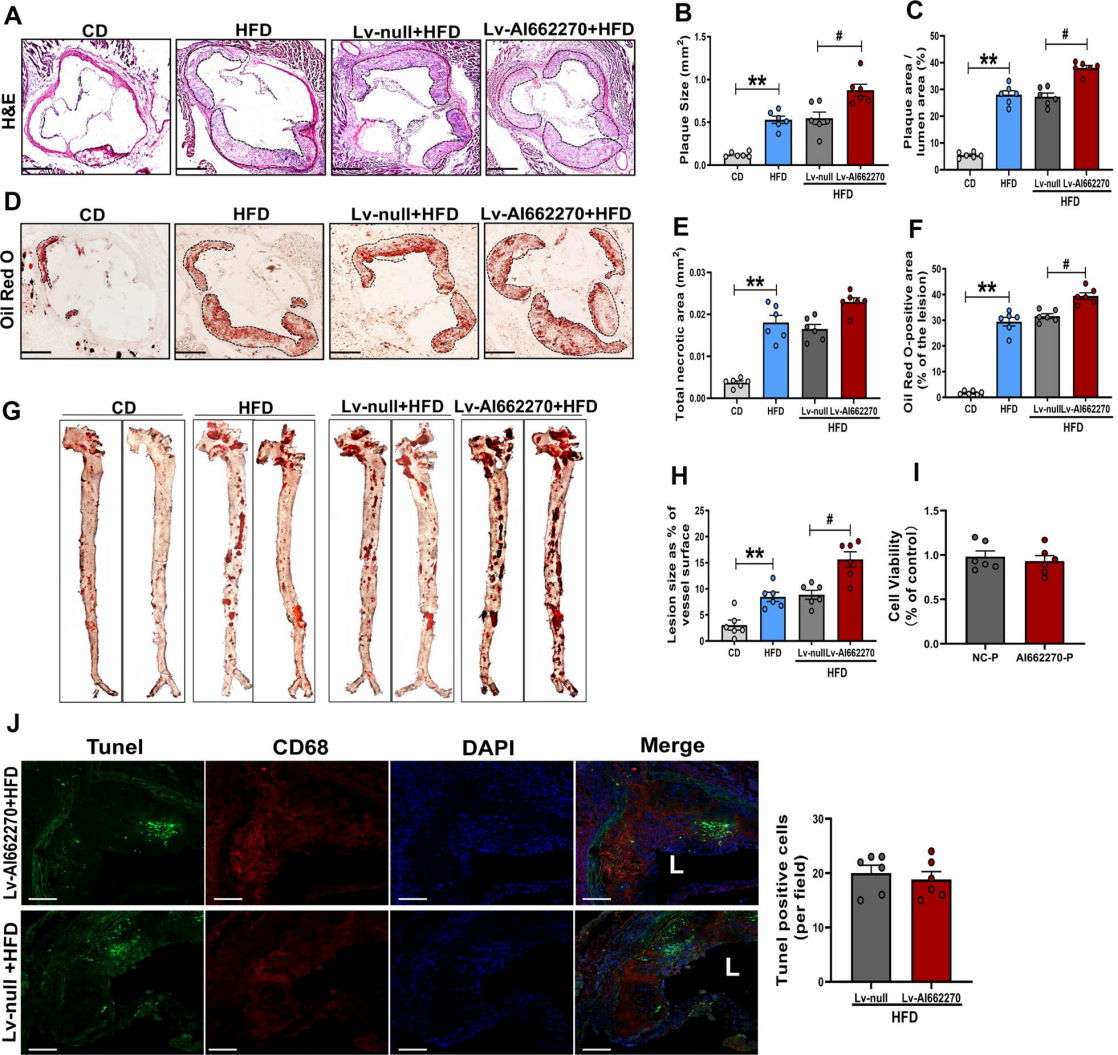
AI662270 increases atherosclerotic lesion size and arrests lipid degradation during atherogenesis. **A-F** Representative images of aortic root cross sections stained with hematoxylin and eosin (H&E) (**A**) and analysis (**B**, **C**, **E**), and oil red O (**D**) and analysis (**F**) from *ApoE*^*−/−*^ mice intravenously injected with Lv-AI662270 and Lv-null or saline fed with HFD or CD for 16 weeks (Scale bar = 400 μm). **G-H** Representative oil red O staining of aortas and analysis of *ApoE*^*−/−*^ mice injected with Lv-AI662270 and Lv-null or saline fed with HFD or CD for 16 weeks. **I** CCK-8 assay was performed to measure the effect of AI662270 on macrophages viability. **J** Tunel positive assay was performed from *ApoE*^*−/−*^ mice intravenously injected with Lv-AI662270 and Lv-null fed with HFD for 16 weeks (Scale bar = 20 μm), “L” represents lumen. n = 6 mice, each dot denotes an individual animal. **p < 0.01 *vs.* mice in CD. #*p* < 0.05 *vs.* Lv-null treated mice in HFD. Data are expressed as mean ± SEM

To investigate the role of AI662270 in regulating atherosclerosis, we evaluated the lesion size of the aortic sinus and *en face* of the aortas. Lv-AI662270 increased the plaque area in total lumen area, but had little effects on necrotic core area in *ApoE*^*−/−*^ mice after 16-weeks of HFD (Fig. 2A–C and E). Enlargement of the oil red O positive staining of the aortic sinus infected by Lv-AI662270 was evident (Fig. 2D, F). Substantial increases in atherosclerotic lesions were also observed in the *en face* of aortas in *ApoE*^*−/−*^ mice infected with Lv-AI662270 following the 16-week HFD (Fig. 2G, H). Overexpression of AI662270 had little effects on macrophages viability in vitro and apoptosis in vivo (Fig. 2I, J). Moreover, we found that overexpression of AI662270 promoted cardiac dysfunction in *ApoE*^*−/−*^ mice fed with HFD, mainly manifested as the decrease of EF and FS, and the increase of HR, systolic LVID (LVID, s), but not diastolic LVID (LVID, d), systolic LVPW (LVPW, s) and diastolic LVPW (LVPW d) (Table 4).

**Table 4:** AI662270 promotes cardiac function decline in *ApoE*^−/−^ mice with high fat diet (HFD)

	CD	HFD	Lv-null + HFD	Lv-AI662270 + HFD
EF (%)	69.62 ± 4.32	48.72 ± 7.08***	52.89 ± 6.57	34.97 ± 3.17^**##**^
FS (%)	38.14 ± 3.25	23.63 ± 5.08***	23.93 ± 3.66	16.58 ± 1.50^**#**^
LVPW, d (mm)	0.83 ± 0.29	0.93 ± 0.28	0.93 ± 0.28	0.66 ± 0.17
LVPW, s (mm)	1.18 ± 0.32	1.28 ± 0.46	1.30 ± 0.27	0.82 ± 0.22
LVID, d (mm)	3.17 ± 0.37	3.74 ± 0.73	3.73 ± 0.70	4.47 ± 0.75
LVID, s (mm)	1.97 ± 0.31	3.05 ± 0.52**	2.84 ± 0.56	3.90 ± 0.45^**#**^
HR, (bmp)	507.83 ± 8.43	564.17 ± 3.75**	573.5 ± 11.2	606.17 ± 6.04^**##**^

n = 6. **p < 0.01 or ***p < 0.001 *vs.* mice in chow diet (CD); #*p* < 0.05 or ##*p* < 0.01 *vs.* Lv-null treated mice in high fat diet (HFD). Data are expressed as mean ± SEM

### Effects of AI662270 on foam cell formation, number of macrophages, and collagen content in *ApoE*^*−/−*^ mice

**Fig. 3:**
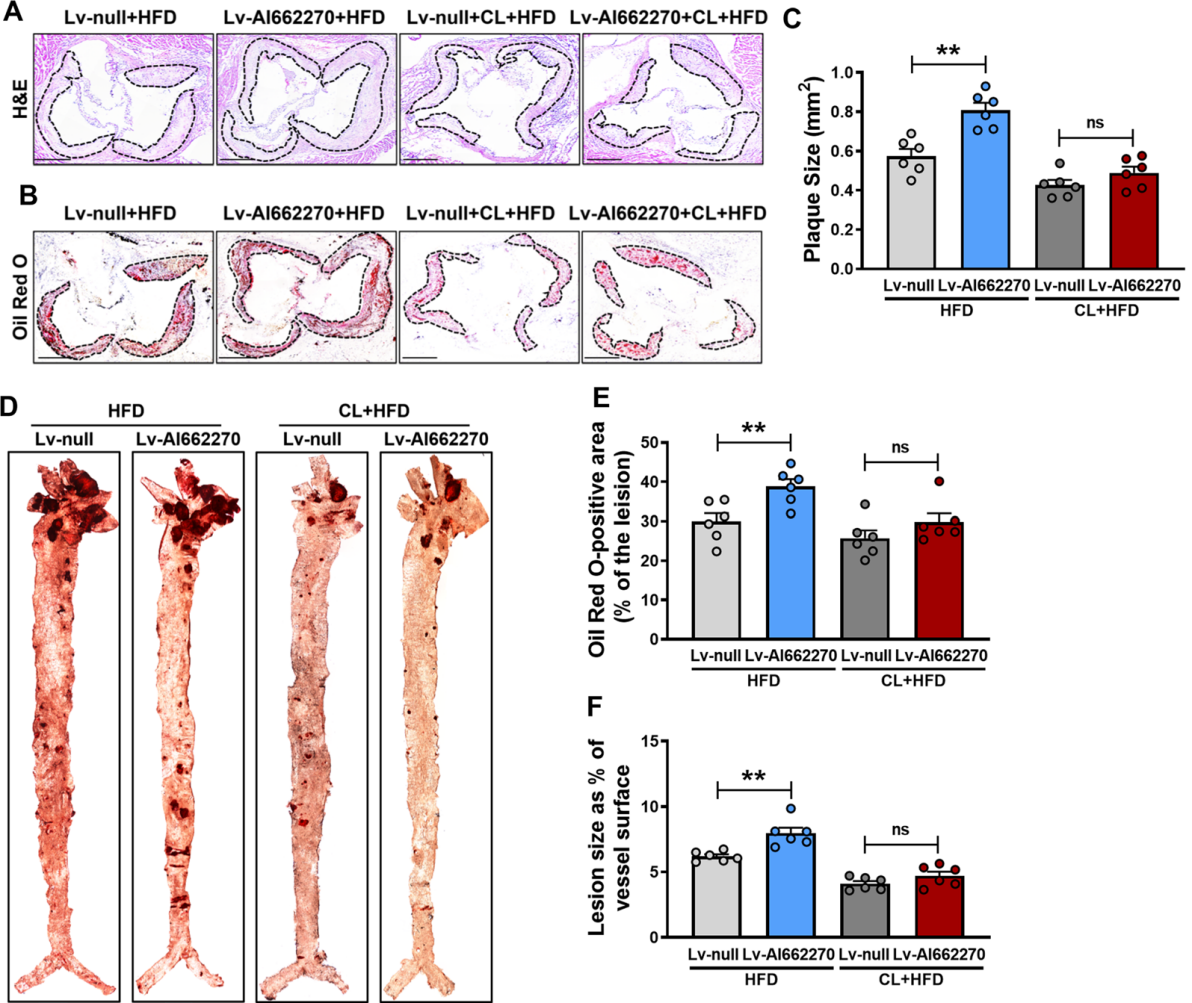
The role of AI662270 in promoting atherosclerosis progression was eliminated by macrophage clearance. **A**–**C, E** Representative images of aortic root cross sections stained with H&E (**A**) and analysis (**C**), and oil red O (**B**) and analysis (**E**) from *ApoE*^*−/−*^ mice intravenously injected with Lv-AI662270 and Lv-null or saline, with or without liposomal clondronate (LC), fed with HFD for 16 weeks (Scale bar = 400 μm). **D, F** Representative oil red O staining of aortas (**D**) and analysis (**F**) of *ApoE*^*−/−*^ mice injected with Lv-AI662270 and Lv-null or saline,with or without LC, fed with HFD for 16 weeks. n = 6 mice, each dot denotes an individual animal. **p < 0.01 *vs.* mice in Lv-null treated mice in HFD, “ns” means no significance. Data are expressed as mean ± SEM

Based on the fact that dysfunction of cholesterol metabolism in macrophages is a crucial event in the early stages of atherosclerotic lesions [20], we evaluated whether AI662270 promotes atherosclerosis progression through macrophages. The macrophage scavenger CL was used to destroy macrophages, and the effect of AI662270 on plaque formation in atherogenic mice was evaluated. We found that AI662270 had no significant effect on plaque area (Fig. 3A, C) and lipid deposition (Fig. 3B, E) of aortic root, and lipid deposition of aorta (Fig. 3D, F) after macrophage clearance in atherosclerotic mice. These results suggest that AI662270 is dependent on macrophages to play a pro-atherogenic role. Therefore, we further explore the regulatory effect of AI662270 on macrophages in the process of atherosclerosis.

**Fig. 4:**
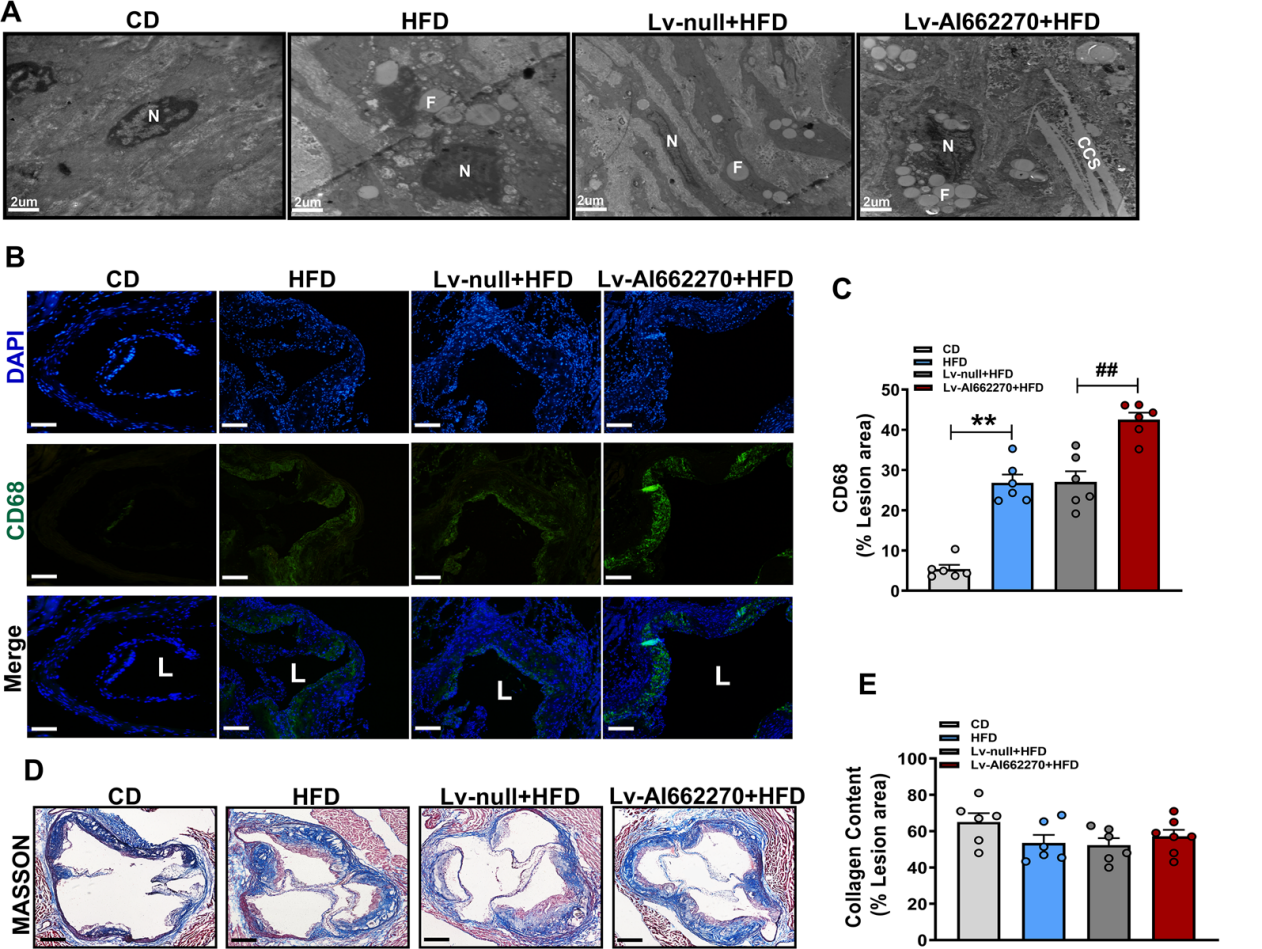
Effect of AI662270 on formation of foam cells, macrophages, and collagen content. **A** Foam cells was enriched in lesion (Scale bar = 2 μm, n = 3 mice). “N” represents nuclei; “F” represents foam cells; “CCS” represents cholesterol crystals. **B**, **C** The macrophage content was denoted by probing with CD68 (Scale bar = 20 μm, n = 6 mice), “L” represents lumen. **D**, **E** Collagen content was analyzed via MASSON staining (Scale bar = 400 μm, n = 6 mice); **p < 0.01 *vs.* mice in CD, ##*p* < 0.01 *vs.* Lv-null treated mice in HFD. Data are expressed as mean ± SEM

Transmission electron microscopy was performed to analyze the formation of foam cells in lesions. Compared with the Lv-null group, more foam cells, characterized by abundant, large lipid droplets, were observed in HFD-fed mice infected with Lv-AI662270, as shown in Fig. 4A. Immunofluorescence staining analysis of mouse aortic sinus plaques revealed that the CD68-positive areas were considerably increased in Lv-AI662270 treated mice (Fig. 4B, C). Atherosclerotic plaques in the Lv-AI662270 and Lv-null infected groups of *ApoE*^*−/−*^ mice contained similar amounts of collagen (Fig. 4D, E). These findings indicated that AI662270 aggravated atherosclerosis by promoting foam cell formation and by increasing the number of macrophages in lesion, but probably had no influence on plaque stability.

### AI662270 mitigates Abca1 function and expression by directly targeting Abca1

**Fig. 5:**
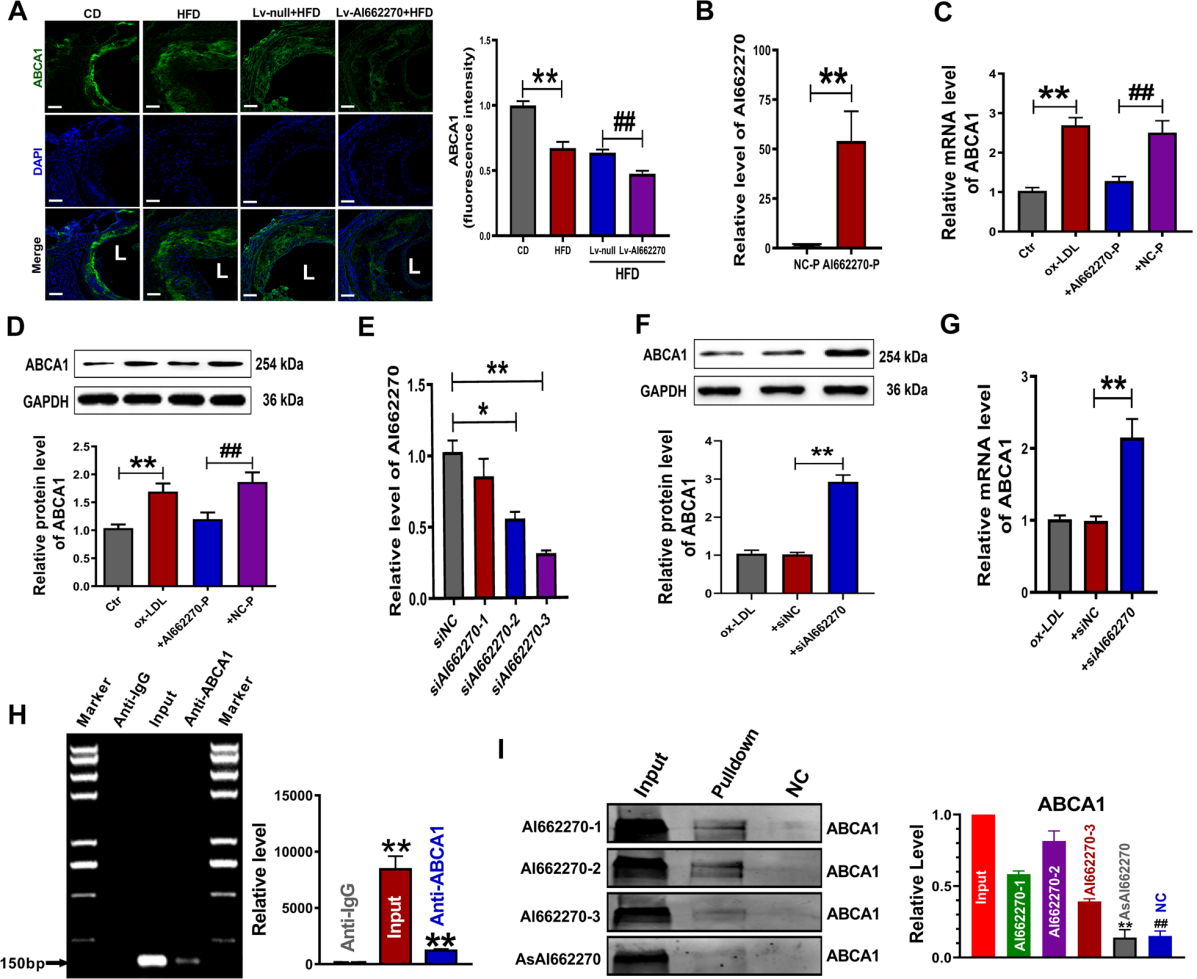
AI662270 regulates its direct target Abca1. **A** Representative examples of cross sections from aortic sinus labeled for Abca1, and Abca1 fluorescence intensity was analyzed (Scale bar = 20 µm, n = 4 mice); **p < 0.01 *vs.* mice in CD; ##*p* < 0.01 *vs.* Lv-null treated mice in HFD. **B** Transfection efficacy was measured using qRT-PCR (n = 5); **p < 0.01 *vs*. NC-P. **C**, **D** Relative mRNA and protein expression of Abca1 were determined derived from AI662270 overexpression in peritoneal macrophages treated with ox-LDL (100 µg/mL). n = 6 in (**C**) and n = 5 in (**D**); **p < 0.01 *vs.* Ctr; ##*p* < 0.01 *vs.* + NC-P. **E** Transfection efficacy was detected using qRT-PCR (n = 6); *p < 0.05 or **p < 0.01 *vs*. + siNC. **F, G** Protein (**F**) (n = 3) and mRNA (**G**) (n = 6) expression of Abca1 were analyzed. **p < 0.01 *vs*. + siNC. “ + ” represents peritoneal macrophages cotreatment with ox-LDL (100 µg/mL) in (**C**, **D**, **F**, **G**). **H** RNA-interacting protein immunoprecipitation (RIP) analysis for AI662270:Abca1 interaction (n = 3); **p < 0.01 *vs.* anti-IgG. **I** RNA pulldown of AI662270 dragged down an appreciable quantity of Abca1 (n = 3). **p < 0.01*,* ##*p* < 0.01 *vs.* AI662270-1/2. Data are expressed as mean ± SEM

Restricting ATP-binding cassette transporter Abca1 expression reportedly arrests Abca1-mediated cholesterol efflux, thereby promoting foam cell formation [28]. We first determined the potential regulatory role of AI662270 in Abca1. Immunofluorescence results revealed that Lv-AI662270 treatment notably decreased Abca1 levels in the aortic root (Fig. 5A). Examination of peritoneal macrophages verified the overexpression efficiency of AI662270 (> 50-fold increase) after transfection with plasmid AI662270-P carrying the AI662270 gene (Fig. 5B). Abca1 levels were obviously increased in mRNA and protein samples isolated from peritoneal macrophages incubated with ox-LDL. However, the mRNA and protein levels of Abca1 were dominantly repressed upon overexpression of AI662270 (Fig. 5C, D). Moreover, three siRNAs against AI662270 (siAI662270) were used to further demonstrate their regulatory role in Abca1 activation. SiAI662270-3 remarkably reduced endogenous AI662270 by approximately 60% (Fig. 5E), and was therefore selected for subsequent experiments. AI662270 knockdown increased both the protein level and mRNA level of Abca1 (Fig. 5F, G). To determine whether AI662270 can directly affect the expression of Abca1 and regulate its function, we performed a theoretical analysis on RNA:protein binding using RNA–Protein Interaction Prediction (RPISeq) database (http://pridb.gdcb.iastate.edu/RPISeq/). Our analysis predicted a high probability of AI662270:Abca1 interaction (Additional file 4: Fig. S3 and Additional file 5: Fig. S4). Thus, RIP assay was performed to explore whether AI662270 could physically bind to Abca1. Immunoprecipitation of Abca1 included an appreciable amount of AI662270 (Fig. 5H). Consistently, RNA pulldown of AI662270 included an appreciable quantity of Abca1. Abca1 was specifically enriched by the probe AI662270-1/2 (Fig. 5I). Both RIP and RNA pulldown experiments proved that AI662270 has a strong affinity with Abca1. Furthermore, we found that overexpression of AI662270 decreased the level of scavenger receptor class B, member I (SR-BI), while knockdown of AI662270 increased the expression of SR-BI, but had no significant effect on Abcg1 and CD36 (Additional file 3: Fig. S2I, J). Moreover, the result revealed that AI662270 had little effect on macrophage inflammatory status/phenotypes and the development of atherosclerosis in CD-fed *ApoE*^*−/−*^ mice (Additional file 3: Fig. S2K–M).

### AI662270 limits cholesterol efflux and promotes foam cell formation in peritoneal macrophages

**Fig. 6:**
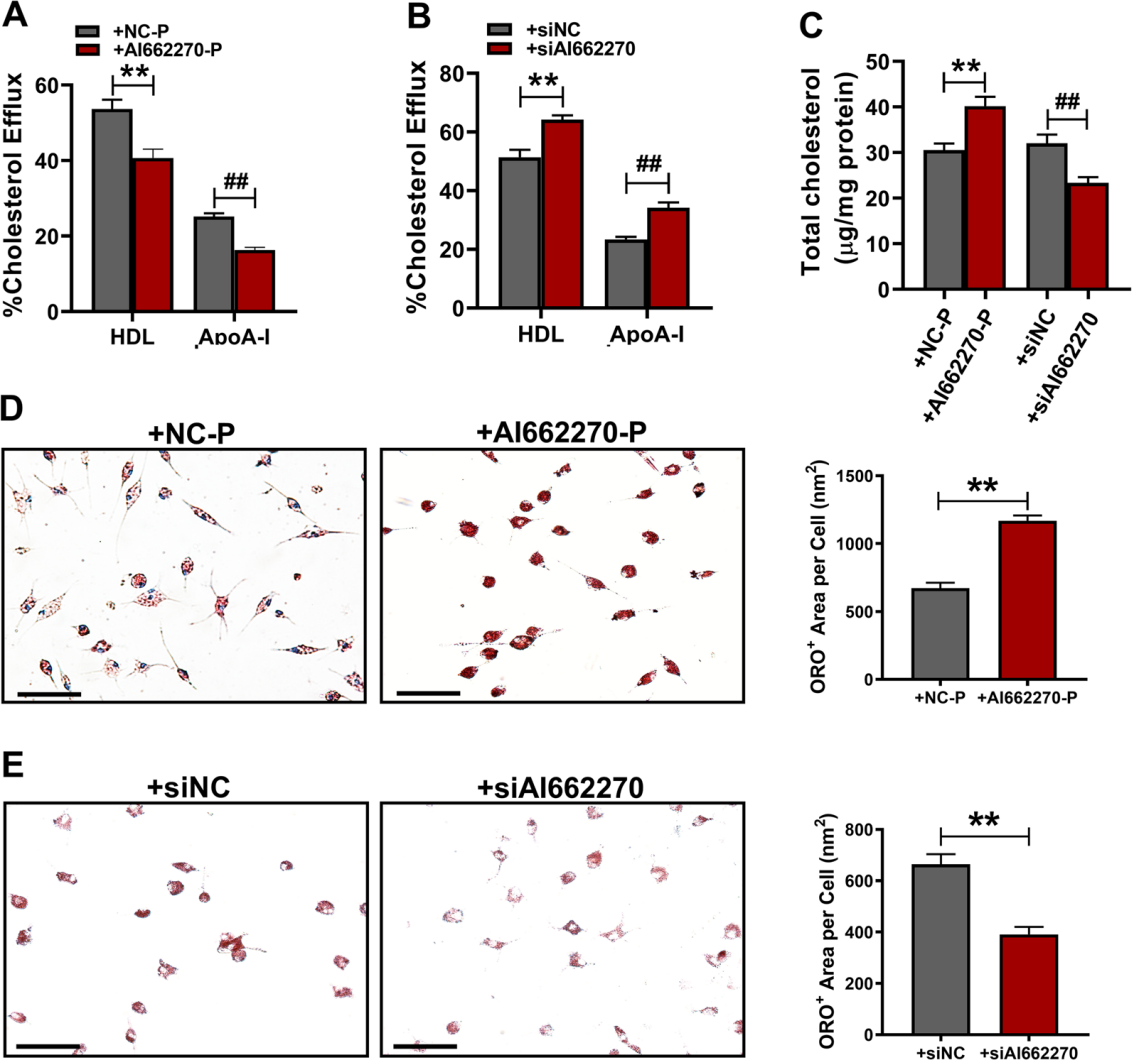
AI662270 initiates cholesterol efflux and promotes foam cell formation. **A**, **B** Cholesterol efflux assay kit was used to measure cholesterol efflux to HDL or ApoA-I in peritoneal macrophages isolated from + AI662270-P (**A**) or + siAI662270 (**B**) (n = 5). **C** The total cholesterol content was assessed with a coupled enzyme assay (n = 5); **p < 0.01 *vs.* + NC-P, ##*p* < 0.01 *vs.* + siNC. **D**, **E** Oil red O staining was performed to verify the effect of AI662270 on foam cell formation in + AI662270-P (**D**) or + siAI662270 (**E**) (Scale bar = 100 µm, n = 5); **p < 0.01 *vs.* + NC-P or + siNC. “ + ” represents peritoneal macrophages cotreatment with ox-LDL (100 µg/mL). Data are expressed as mean ± SEM

The above results indicated the important role of AI662270 in regulating Abca1. However, whether AI662270 directly affects cholesterol efflux and contributes to foam cell formation still unknown. To assess the physiological significance of Abca1 regulation by AI662270, we performed cholesterol efflux assays in peritoneal macrophages. Peritoneal macrophages were transfected with AI662270-P or siAI662270 for 48 h, and treated with either HDL (50 µg/mL) or apolipoprotein A-I (ApoA-I, 25 µg/mL) for 6 h. As shown in Fig. 6A, the macrophage-specific cholesterol efflux to lipid-poor HDL or ApoA-I capacity was significantly decreased by forced expression of AI662270 in vitro. In contrast, knockdown of AI662270 enhanced cholesterol efflux to lipid-poor HDL or ApoA-I (Fig. 6B). Moreover, an intracellular cholesterol assay indicated that the TC content was increased in AI662270-P transfected peritoneal macrophages. In contrast, endogenous AI662270 was decreased by transfecting with siRNA against AI662270, accompanied by decreased TC content (Fig. 6C). Oil red O staining revealed that forced expression of AI662270 significantly enhanced lipid accumulation in peritoneal macrophages (Fig. 6D). In sharp contrast, AI662270 knockdown diminished lipid content (Fig. 6E). These results indicate that AI662270 promotes atherogenesis partially by binding to its direct target Abca1, thereby limiting the functional role of Abca1 and promoting the formation of foam cells.

## Discussion

The present study identifies the highly expressed lncRNA AI662270 as a critical regulator of atherosclerosis. We find that forced expression of the novel AI662270 accelerates the progression of atherosclerosis, characterized by decreased cholesterol efflux and enhanced foam cell formation. Mechanically, partially but at the least, AI662270 attenuates Abca1 expression and activity by directly binding to Abca1, and reduces the SR-BI expression. Moreover, we uncover that AI662270 impairs the cardiac function by attenuating EF and FS, while has no effect on Abcg1/CD36 expression, viability/apoptosis and polarization in macrophages. We also observe that the detrimental effects of AI662270 has no influenced on macrophage-cleared mice, in addition to the expression of eNOS/iNOS expression in vascular endothelium, suggesting that AI662270 depends on macrophages to promote atherosclerosis.

Multiple lncRNAs have been demonstrated to be implicated in regulating the pathophysiological process of macrophages in atherogenesis [29, 30]. LncRNA PELATON is a monocyte- and macrophage-specific lncRNA, which is upregulated in unstable atherosclerotic plaque. And its knockdown could mitigate macrophages uptake of ox-LDL [31]. LncRNA SNHG16 could negatively regulate nuclear factor-kappa B function to promote the proliferation and inflammatory response of macrophages [32]. LncRNA MIAT positively modulates the expression of the anti-phagocytic molecule CD47 and limits the clearance of apoptotic cells by macrophages, thereby aggravating atherosclerosis [15]. Our findings indicate that the novel lncRNA AI662270 can interact with anti-atherosclerotic protein Abca1 to aggravate atherosclerosis.

Apoptotic and pro-inflammatory macrophages accumulate in lesions and play a detrimental role in the development atherosclerosis [33, 34]. Our results show that AI662270 has no influenced on macrophages viability and apoptosis. Similarly, the pro-inflammatory macrophage (M1) and anti-inflammatory macrophage (M2) are also not altered by the dysregulated AI662270. In addition to macrophages, vascular endothelial cells, smooth muscle cells and fibroblasts play crucial role in the development of atherosclerosis [35, 36]. We find that AI662270 mainly expresses in macrophages, but not in endothelial cells, smooth muscle cells and fibroblasts. Further experiments indicate that forced expression of AI662270 has no influenced on the eNOS and iNOS in MAECs. Excess cholesterol accumulation in macrophages favoring foam cell formation is one of the main causes of atherosclerosis [5]. Importantly, the pro-atherogenic effect of AI662270 is virtually eliminated after macrophage clearance with macrophage scavenger CL, which further clarifies that AI662270 regulates the progression of atherosclerosis mainly by affecting the function of macrophages. Due to the influence of tissue-resident macrophages [37], smooth muscle cells-derived macrophages [38], etc., oil red O staining in aorta and roots remained partially positive after the application of macrophage scavenger CL. Abca1, a member of the ABC transmembrane transporter family, is a critical cholesterol transporter that removes excess cellular cholesterol to lipid-poor ApoA-I and ApoE to form HDL particles [39, 40]. We find that AI662270 directly binds to Abca1 protein, thereby inhibiting the expression of Abca1 and limiting cholesterol efflux. In this regard, we also observe that overexpression of AI662270 induces foam cell formation, whereas its knockdown produces the opposite effect. Together, these results indicate that AI662270 aggravates atherosclerosis, at least partially, by impairing the function of Abca1 and inhibiting the SR-BI expression.

In summary, our results shed light on the critical role of macrophage AI662270 during atherogenesis. Forced expression of AI662270 promoted foam cell formation by acting as an Abca1 inhibitor and repressing the SR-BI expression, accelerating the progression of atherosclerosis. Moreover, these effects accounted for the adverse lipid accumulation in plasma and the impaired cardiac function observed in mice with AI662270 overexpression.

## Supplementary Information

**Additional file 1.** Additional methods and results section.

**Additional file 2: Fig. S1. **Expression and effect of AI662270 in different cell types. **A** The expression of AI662270 in different cells treated with ox-LDL (100 µg/mL) was determined by qRT-PCR (n=4). *p<0.05, ###*p*<0.001 *vs.* Control (Ctr) in macrophage. **B-C** Immunofluorescence staining of eNOS (**B**) (Scale bar=20 μm, n=3 mice), and iNOS (**C**) (Scale bar=25 μm, n=3 mice) in the aortic root was performed, and the vascular endothelium was labeled with CD31, “L” represents lumen. **D-E **Relative protein expression of eNOS (**D**) and iNOS (**E**) were determined derived from AI662270 overexpression in MAECs treated with ox-LDL (100 µg/mL) (n=5). Data are expressed as mean±SEM.

**Additional file 3: Fig. S2.** Functional role of AI662270 on lipid and lipoprotein profile, body weight, and glucose metabolism in *ApoE*^*-*/-^ mice. **A-D** Quantification of total cholesterol (TC) (**A**), low density lipoprotein cholesterol (LDL-C) (**B**), high-density lipoprotein cholesterol (HDL-C) (**C**), triglyceride (TG) (**D**) from Lv-null and Lv-AI662270 mice fed with CD or HFD for 16 weeks (n=6 mice); **p<0.01 *vs.* mice in CD; ##*p*<0.01 *vs.* Lv-null treated mice in HFD. **E-G** Body weight (BW) (**E**), insulin tolerance test (ITT) (**F**), and glucose tolerance test (GTT) (**G**) analyses of Lv-AI662270 and Lv-null mice fed with HFD were performed (n=6 mice); *p<0.05 *vs.* Lv-null treated mice in HFD. **H** Fed and fasted blood glucose levels in Lv-null and Lv-AI662270 mice (n=6 mice).** I, J** The expression of Abca1, Abcg1, SR-BI and CD36 were detected by using qRT-PCR. (n=6); *p<0.05 *vs.* NC-P+ or siNC+. “+” denotes ox-LDL (100 µg/mL incubation for 12 h). **K, L** The expression of TNF-α, IL-1β, ARG-1 and Retnla were measured by using qRT-PCR (n=6). “+” denotes LPS (100 ng/mL) + IFN-γ (15 ng/mL) incubation for 24 h in (K); “+” denotes IL-4 (20 ng/mL) incubation for 24 h in (L). **M** Hematoxylin and eosin (H&E) from *ApoE*^*-/-*^ mice intravenously injected with Lv-AI662270 and Lv-null fed with CD for 16 weeks (Scale bar=400 μm, n=6). Data are expressed as mean±SEM.

**Additional file 4: Fig. S3.** Computational analysis for RNA:protein binding using RPISeq database suggesting a high probability of AI662270:Abca1 interaction.

**Additional file 5:** **Fig. S4.** Computational analysis for RNA:protein binding using catRAPID database to design the probe sequence of AI662270-1/-2/-3.

## Data Availability

The datasets generated and/or analysed during the current study are available in the manuscript and additional materials.

## Abbreviations

Abca1: Adenosine triphosphate-binding cassette transporter A1
ApoA-I: Apolipoprotein A-I
ApoE: Apolipoprotein E
CCK-8: Cell counting kit-8
CD: Chow diet
EF: Ejection fraction
eNOS: Endothelial NOS
FBS: Fetal bovine serum
FS: Fractional shortening
GAPDH: Glyceraldehyde 3-phosphate dehydrogenase
GTT: Glucose tolerance test
H&E: Hematoxylin and eosin
HDL: High density lipoprotein
HFD: High fat diet
HR: Heart rate
i.p.: Intraperitoneal
iNOS: Inducible nitric oxide synthase
ITT: Insulin tolerance test
LC: Liposomal clondronate
LncRNAs: Long noncoding RNAs
LVID: Left ventricular internal dimension
LVPW: Left ventricular posterior wall
MAECs: Mouse aortic endothelial cells
MOVAS: Mouse aortic vascular smooth muscle cells
OCT: Optimal cutting temperature compound
ox-LDL: Oxidized low density lipoprotein
PBS: Phosphate buffered saline
PFA: Paraformaldehyde
qRT-PCR: Quantitative real-time reverse transcription polymerase chain reaction
RIP: RNA-interacting protein immunoprecipitation
siRNA: Small interfering RNA
SR-BI: Scavenger receptor class B, member I
TC: Total cholesterol
TG: Triglyceride

## References

[1] Huang L, Chambliss KL, Gao X, Yuhanna IS, Behling-Kelly E, Bergaya S (2019). SR-B1 drives endothelial cell LDL transcytosis via DOCK4 to promote atherosclerosis. Nature.

[2] Weber C, Noels H (2011). Atherosclerosis: current pathogenesis and therapeutic options. Nat Med.

[3] Glass CK, Witztum JL (2001). Atherosclerosis. the road ahead. Cell.

[4] Koelwyn GJ, Corr EM, Erbay E, Moore KJ (2018). Regulation of macrophage immunometabolism in atherosclerosis. Nat Immunol.

[5] Moore KJ, Sheedy FJ, Fisher EA (2013). Macrophages in atherosclerosis: a dynamic balance. Nat Rev Immunol.

[6] Shah PK, Falk E, Badimon JJ, Fernandez-Ortiz A, Mailhac A, Villareal-Levy G (1995). Human monocyte-derived macrophages induce collagen breakdown in fibrous caps of atherosclerotic plaques. Potential role of matrix-degrading metalloproteinases and implications for plaque rupture. Circulation.

[7] Westerterp M, Murphy AJ, Wang M, Pagler TA, Vengrenyuk Y, Kappus MS (2013). Deficiency of ATP-binding cassette transporters A1 and G1 in macrophages increases inflammation and accelerates atherosclerosis in mice. Circ Res.

[8] Yvan-Charvet L, Ranalletta M, Wang N, Han S, Terasaka N, Li R (2007). Combined deficiency of ABCA1 and ABCG1 promotes foam cell accumulation and accelerates atherosclerosis in mice. J Clin Invest.

[9] Rayner KJ, Suarez Y, Davalos A, Parathath S, Fitzgerald ML, Tamehiro N (2010). MiR-33 contributes to the regulation of cholesterol homeostasis. Science.

[10] Fatica A, Bozzoni I (2014). Long non-coding RNAs: new players in cell differentiation and development. Nat Rev Genet.

[11] Yang L, Li P, Yang W, Ruan X, Kiesewetter K, Zhu J (2016). Integrative transcriptome analyses of metabolic responses in mice define pivotal LncRNA metabolic regulators. Cell Metab.

[12] Geisler S, Coller J (2013). RNA in unexpected places: long non-coding RNA functions in diverse cellular contexts. Nat Rev Mol Cell Biol.

[13] Cremer S, Michalik KM, Fischer A, Pfisterer L, Jae N, Winter C (2019). Hematopoietic deficiency of the long noncoding RNA MALAT1 promotes atherosclerosis and plaque inflammation. Circulation.

[14] Sallam T, Jones M, Thomas BJ, Wu X, Gilliland T, Qian K (2018). Transcriptional regulation of macrophage cholesterol efflux and atherogenesis by a long noncoding RNA. Nat Med.

[15] Ye ZM, Yang S, Xia YP, Hu RT, Chen S, Li BW (2019). LncRNA MIAT sponges miR-149-5p to inhibit efferocytosis in advanced atherosclerosis through CD47 upregulation. Cell Death Dis.

[16] Fan Y, Zhang Y, Zhao H, Liu W, Xu W, Jiang L (2023). lncR-GAS5 upregulates the splicing factor SRSF10 to impair endothelial autophagy, leading to atherogenesis. Front Med.

[17] Arvaniti E, Moulos P, Vakrakou A, Chatziantoniou C, Chadjichristos C, Kavvadas P (2016). Whole-transcriptome analysis of UUO mouse model of renal fibrosis reveals new molecular players in kidney diseases. Sci Rep.

[18] Fukuda M, Sakaue-Sawano A, Shimura C, Tachibana M, Miyawaki A, Shinkai Y (2019). G9a-dependent histone methylation can be induced in G1 phase of cell cycle. Sci Rep.

[19] Shoulders H, Garner KH, Singla DK (2019). Macrophage depletion by clondronate attenuates BMP-7 induced M2 macrophage differentiation and improved systolic blood velocity in atherosclerosis. Transl Res.

[20] Ulrich V, Rotllan N, Araldi E, Luciano A, Skroblin P, Abonnenc M (2016). Chronic miR-29 antagonism promotes favorable plaque remodeling in atherosclerotic mice. EMBO Mol Med.

[21] Canfran-Duque A, Rotllan N, Zhang X, Fernandez-Fuertes M, Ramirez-Hidalgo C, Araldi E (2017). Macrophage deficiency of miR-21 promotes apoptosis, plaque necrosis, and vascular inflammation during atherogenesis. EMBO Mol Med.

[22] Fan Y, Liu L, Fang K, Huang T, Wan L, Liu Y (2016). Resveratrol ameliorates cardiac hypertrophy by down-regulation of miR-155 through activation of breast cancer type 1 susceptibility protein. J Am Heart Assoc.

[23] Zheng X, Yu Q, Shang D, Yin C, Xie D, Huang T (2022). TAK1 accelerates transplant arteriosclerosis in rat aortic allografts by inducing autophagy in vascular smooth muscle cells. Atherosclerosis.

[24] Edgar R, Domrachev M, Lash A (2002). Gene expression omnibus: NCBI gene expression and hybridization array data repository. Nucleic Acids Res.

[25] Guo X, Gao L, Liao Q, Xiao H, Ma X, Yang X (2013). Long non-coding RNAs function annotation: a global prediction method based on bi-colored networks. Nucleic Acids Res.

[26] Liu W, Bai X, Liu Y, Wang W, Han J, Wang Q (2015). Topologically inferring pathway activity toward precise cancer classification via integrating genomic and metabolomic data: prostate cancer as a case. Sci Rep.

[27] Zhao Y, Li H, Fang S, Kang Y, Wu W, Hao Y (2016). NONCODE 2016: an informative and valuable data source of long non-coding RNAs. Nucleic Acids Res.

[28] Tumurkhuu G, Dagvadorj J, Porritt RA, Crother TR, Shimada K, Tarling EJ (2018). *Chlamydia pneumoniae* hijacks a host autoregulatory IL-1beta loop to drive foam cell formation and accelerate atherosclerosis. Cell Metab.

[29] Zhang Z, Salisbury D, Sallam T (2018). Long noncoding RNAs in atherosclerosis: JACC review topic of the week. J Am Coll Cardiol.

[30] Aryal B, Suarez Y (2019). Non-coding RNA regulation of endothelial and macrophage functions during atherosclerosis. Vascul Pharmacol.

[31] Hung J, Scanlon JP, Mahmoud AD, Rodor J, Ballantyne M, Fontaine MAC (2020). Novel plaque enriched long noncoding RNA in atherosclerotic macrophage regulation (PELATON). Arterioscler Thromb Vasc Biol.

[32] An JH, Chen ZY, Ma QL, Wang HJ, Zhang JQ, Shi FW (2019). LncRNA SNHG16 promoted proliferation and inflammatory response of macrophages through miR-17-5p/NF-kappaB signaling pathway in patients with atherosclerosis. Eur Rev Med Pharmacol Sci.

[33] Randolph GJ (2013). Proliferating macrophages prevail in atherosclerosis. Nat Med.

[34] Tabas I (2010). Macrophage death and defective inflammation resolution in atherosclerosis. Nat Rev Immunol.

[35] Bijak M, Saluk J, Tsirigotis-Maniecka M, Komorowska H, Wachowicz B, Zaczyńska E (2013). The influence of conjugates isolated from *Matricaria** chamomilla* L. on platelets activity and cytotoxicity. Int J Biol Macromol.

[36] Akoumianakis I, Polkinghorne M, Antoniades C (2022). Non-canonical WNT signalling in cardiovascular disease: mechanisms and therapeutic implications. Nat Rev Cardiol.

[37] Al-Rifai R, Vandestienne M, Lavillegrand J, Mirault T, Cornebise J, Poisson J (2022). JAK2V617F mutation drives vascular resident macrophages toward a pathogenic phenotype and promotes dissecting aortic aneurysm. Nat Commun.

[38] Robichaud S, Rasheed A, Pietrangelo A, Doyoung Kim A, Boucher DM, Emerton C (2022). Autophagy is differentially regulated in leukocyte and nonleukocyte foam cells during atherosclerosis. Circ Res.

[39] Hirsch-Reinshagen V, Burgess BL, Wellington CL (2009). Why lipids are important for Alzheimer disease?. Mol Cell Biochem.

[40] Price NL, Rotllan N, Zhang X, Canfran-Duque A, Nottoli T, Suarez Y (2019). Specific disruption of Abca1 targeting largely mimics the effects of miR-33 knockout on macrophage cholesterol efflux and atherosclerotic plaque development. Circ Res.

