# Supplementary material for: Genetic dissection of the impact of lncRNA AI662270 during the development of atherosclerosis
Source: J Transl Med. 2023 Feb 8;21:97. doi: 10.1186/s12967-023-03962-6 (PMC9906833; doi:10.1186/s12967-023-03962-6)
Supplement: Supplementary file 1 — Additional file 1. Additional methods and results section. [file 12967_2023_3962_MOESM1_ESM.docx]

**Genetic dissection of the impact of lncRNA AI662270 during the development of atherosclerosis**

**Additional file 1. Methods**

**Lipoprotein profile and lipid measurements**

Mice were fasted for 12-14 h before blood samples were obtained by retro-orbital venous plexus puncture. Plasma was separated by centrifugation and stored at -80 °C. Total plasma cholesterol (TC), triglyceride (TG), high-density lipoprotein (HDL), and low-density lipoprotein (LDL) were enzymatically detected according to the manufacturer’s instructions (Nanjing Jiancheng Bioengineering Institute, China).

**Insulin tolerance test (ITT) and glucose tolerance test (GTT)**

ITT in mice was performed following 6 h fasting by [intraperitoneal](file:///C:/Users/86131/Dict/8.10.2.0/resultui/html/index.html#/javascript:;) (i.p.) injection of 0.75 U/kg insulin. Blood glucose measurements were performed using a Contour Ultra blood glucose meter before and 15, 30, 60, and 120 min after insulin injection. GTT was conducted by i.p. injection of 1.5 g/kg glucose. Blood glucose was monitored at 8:00 am after feeding and after fasting for 16 h, and measured at 0, 15, 30, 60, and 120 min after injection.

**Western blot analysis**

Information on primary antibodies as follows, endothelial NOS (eNOS) (32027S, 1:500, Cell Signaling Technology, Boston, MA, USA), inducible nitric oxide synthase (iNOS) (ab178945, 1:1000; Abcam, Cambridge, MA, USA).

**Additional file 1, Results**

**AI662270 is highly expressed in macrophages and has no effect on endothelial cell function**

We found that AI662270 expression was not significantly changed in other cell types, including mouse aortic endothelial cells (MAEC), mouse aortic vascular smooth muscle cells (MOVAS), and mouse fibroblasts L929, except in macrophages after treatment with 100 μg/mL ox-LDL for 12 h. At the same time, compared with macrophages, the expression level of AI662270 in other cell types was quite low (Additional file 2: Fig. S1A). To further verify the effect of AI662270 on endothelial cell function, the protein expression levels of eNOS and iNOS were detected *in* *vivo* and *in vitro*, and it was found that their expression levels were not affected by AI662270 in vascular endothelium of mice fed with HFD (Additional file 2: Fig. S1B-C) and in MAEC treated with ox-LDL (Additional file 2: Fig. S1D-E), indicating that AI662270 had no significant effect on the function of vascular endothelial cells in atherosclerotic mice. In conclusion, AI662270 regulates atherosclerosis progression by affecting macrophage function.

**Forced expression of AI662270 alters lipid profiles *in vivo***

Anomalies in circulating lipid and lipoprotein levels are a critical cause of cardiovascular diseases, especially atherosclerosis [[1](#_ENREF_1), [2](#_ENREF_2)]. Lv-AI662270 infection obviously increased the plasma levels of TC and LDL, and reduced HDL, with no significant effect on TG in *ApoE^-/-^* mice fed with HFD. However, Lv-AI662270 infection had no statistically significant influence on lipid profiles and TG in *ApoE^-/-^* mice fed with CD (Additional file 3: Fig. S2A-D). Moreover, although the results indicated that AI662270 overexpression slightly increased body weight, there was no obvious difference between Lv-AI662270 and Lv-null treated mice throughout the study Additional file 2: Fig. S1E). On the other hand, as revealed by ITT, *ApoE^-/-^* mice infected with Lv-AI662270 and fed with HFD for 16 weeks had elevated glucose concentration and blunted the responsiveness to insulin, suggesting impaired insulin sensitivity (Figure S1F). However, AI662270 overexpression did not significantly affect glucose homeostasis in GTT (Additional file 3: Fig. S2G, H). Furthermore, we found that overexpression of AI662270 decreased the level of scavenger receptor class B, member I (SR-BI), while knockdown of AI662270 increased the expression of SR-BI, but had no significant effect on Abcg1 and CD36 (Additional file 3: Fig. S2I, J). Moreover, the result revealed that AI662270 had little effect on macrophage inflammatory status/phenotypes and the development of atherosclerosis in CD-fed *ApoE^-/-^* mice (Additional file 2: Fig. S1K-M).

**Computational analysis for RNA:protein binding**

Computational analysis for RNA:protein binding using RNA-Protein Interaction Prediction (RPISeq) database suggested a high probability of AI662270*:*Abca1 interaction (Additional file 4: Fig. S3). Moreover, computational analysis for RNA:protein binding using the catRAPID database to design the probe sequence of AI662270-1/-2/-3 (Additional file 5: Fig. S4).

**References:**

1. KONTUSH A, CHAPMAN M J. Functionally defective high-density lipoprotein: a new therapeutic target at the crossroads of dyslipidemia, inflammation, and atherosclerosis [J]. Pharmacol Rev, 2006; 58(3): 342-74.

2. FERNANDEZ-HERNANDO C, ACKAH E, YU J, SUAREZ Y, MURATA T, IWAKIRI Y, et al. Loss of Akt1 leads to severe atherosclerosis and occlusive coronary artery disease [J]. Cell Metab, 2007; 6(6): 446-57.
