# Supplementary figures and images for: Genetic dissection of the impact of lncRNA AI662270 during the development of atherosclerosis
Source: J Transl Med. 2023 Feb 8;21:97. doi: 10.1186/s12967-023-03962-6 (PMC9906833; doi:10.1186/s12967-023-03962-6)

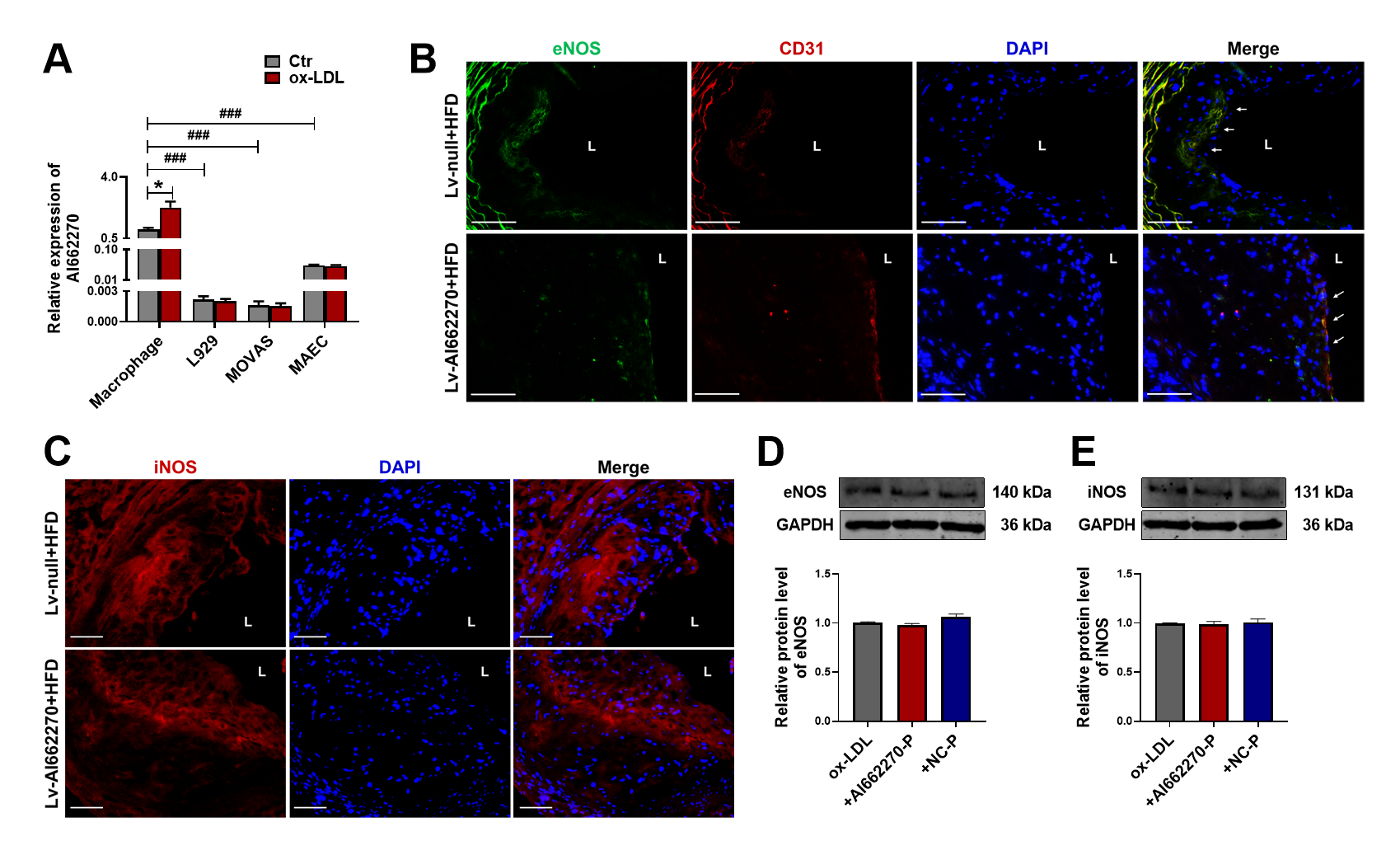

Supplement: Supplementary file 2 — Additional file 2: Fig. S1. Expression and effect of AI662270 in different cell types. A The expression of AI662270 in different cells treated with ox-LDL (100 µg/mL) was determined by qRT-PCR (n=4). *p<0.05, ###p<0.001 vs. Control (Ctr) in macrophage. B-C Immunofluorescence staining of eNOS (B) (Scale bar=20 μm, n=3 mice), and iNOS (C) (Scale bar=25 μm, n=3 mice) in the aortic root was performed, and the vascular endothelium was labeled with CD31, “L” represents lumen. D-E Relative protein expression of eNOS (D) and iNOS (E) were determined derived from AI662270 overexpression in MAECs treated with ox-LDL (100 µg/mL) (n=5). Data are expressed as mean±SEM. [file 12967_2023_3962_MOESM2_ESM.tif]

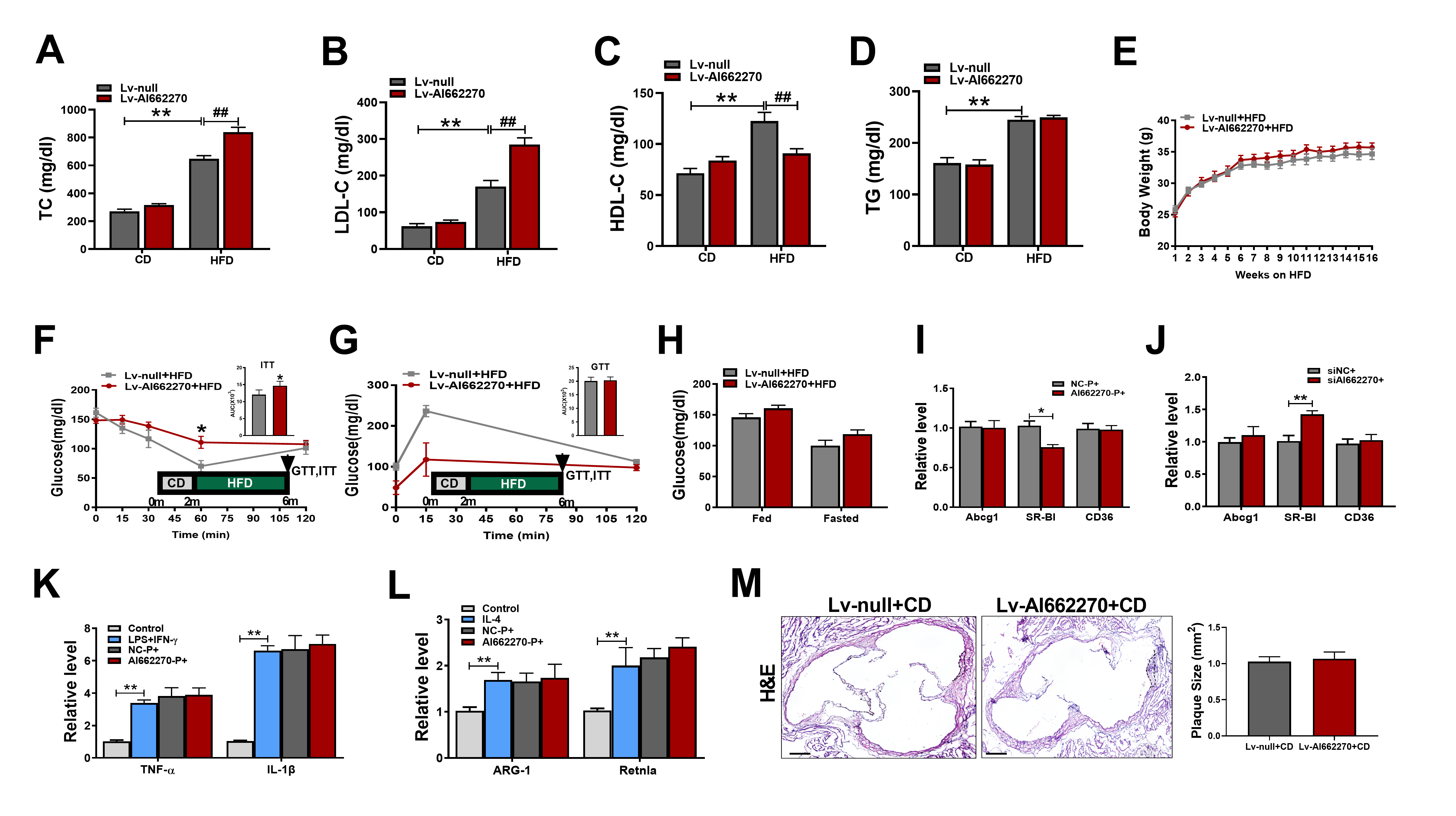

Supplement: Supplementary file 3 — Additional file 3: Fig. S2. Functional role of AI662270 on lipid and lipoprotein profile, body weight, and glucose metabolism in ApoE-/- mice. A-D Quantification of total cholesterol (TC) (A), low density lipoprotein cholesterol (LDL-C) (B), high-density lipoprotein cholesterol (HDL-C) (C), triglyceride (TG) (D) from Lv-null and Lv-AI662270 mice fed with CD or HFD for 16 weeks (n=6 mice); **p<0.01 vs. mice in CD; ##p<0.01 vs. Lv-null treated mice in HFD. E-G Body weight (BW) (E), insulin tolerance test (ITT) (F), and glucose tolerance test (GTT) (G) analyses of Lv-AI662270 and Lv-null mice fed with HFD were performed (n=6 mice); *p<0.05 vs. Lv-null treated mice in HFD. H Fed and fasted blood glucose levels in Lv-null and Lv-AI662270 mice (n=6 mice). I, J The expression of Abca1, Abcg1, SR-BI and CD36 were detected by using qRT-PCR. (n=6); *p<0.05 vs. NC-P+ or siNC+. “+” denotes ox-LDL (100 µg/mL incubation for 12 h). K, L The expression of TNF-α, IL-1β, ARG-1 and Retnla were measured by using qRT-PCR (n=6). “+” denotes LPS (100 ng/mL) + IFN-γ (15 ng/mL) incubation for 24 h in (K); “+” denotes IL-4 (20 ng/mL) incubation for 24 h in (L). M Hematoxylin and eosin (H&E) from ApoE-/- mice intravenously injected with Lv-AI662270 and Lv-null fed with CD for 16 weeks (Scale bar=400 μm, n=6). Data are expressed as mean±SEM. [file 12967_2023_3962_MOESM3_ESM.tif]

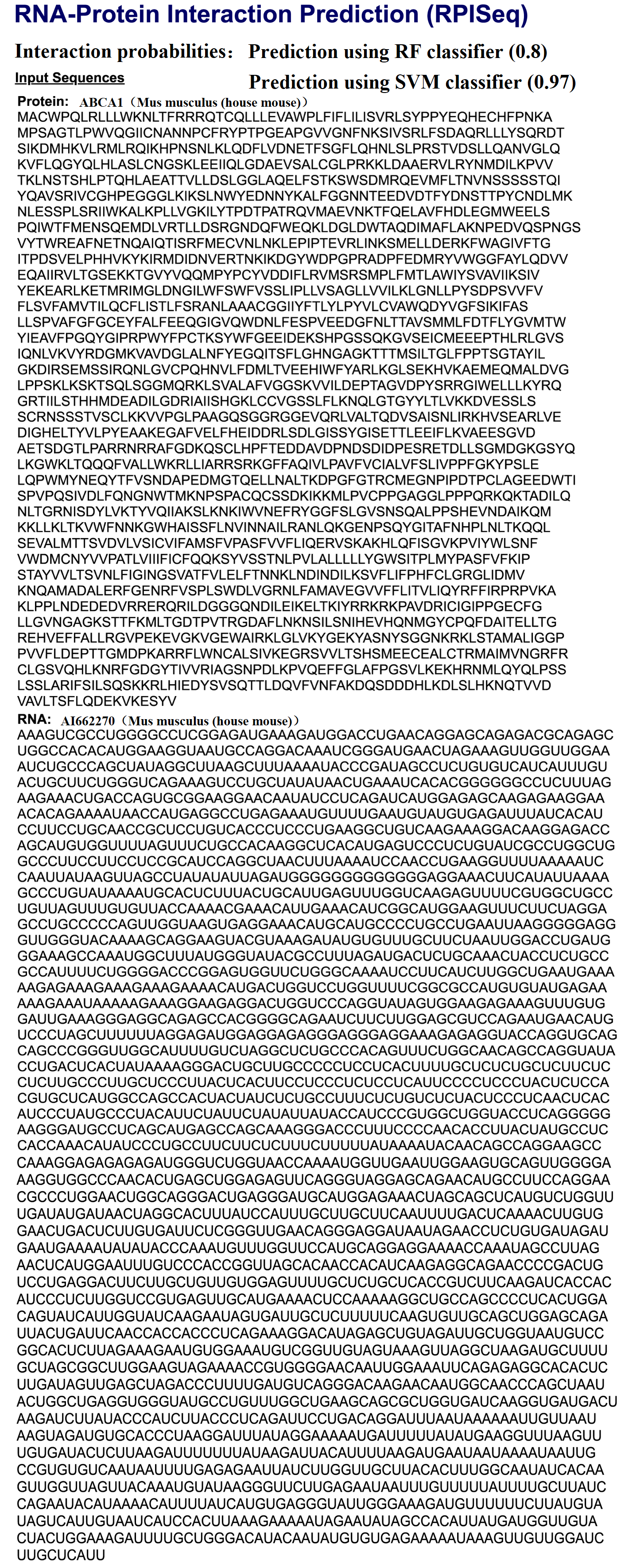

Supplement: Supplementary file 4 — Additional file 4: Fig. S3. Computational analysis for RNA:protein binding using RPISeq database suggesting a high probability of AI662270:Abca1 interaction. [file 12967_2023_3962_MOESM4_ESM.tif]

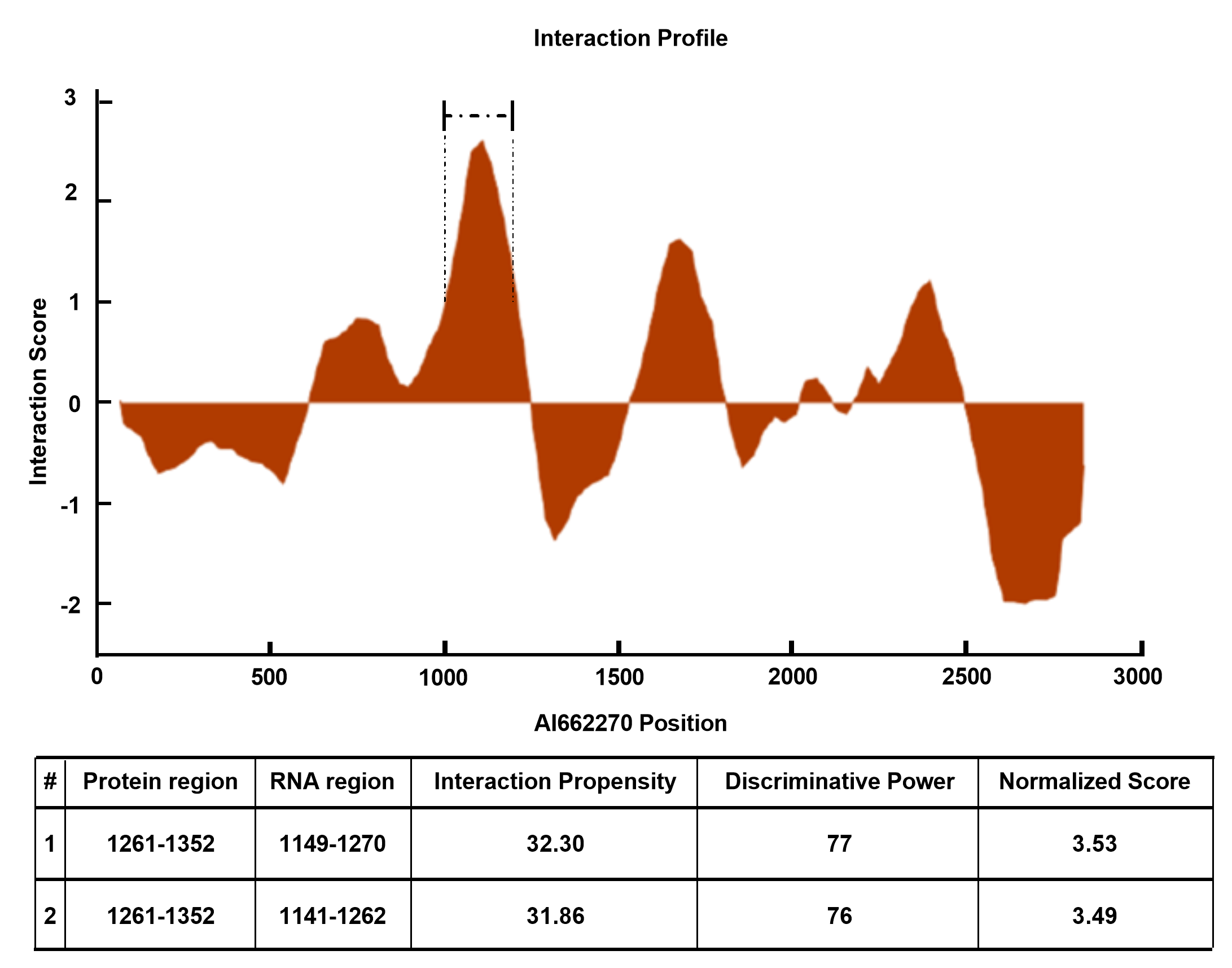

Supplement: Supplementary file 5 — Additional file 5: Fig. S4. Computational analysis for RNA:protein binding using catRAPID database to design the probe sequence of AI662270-1/-2/-3. [file 12967_2023_3962_MOESM5_ESM.tif]
